# Evaluation of residual urine volume after catheterization by ultrasound performed by a nurse

**DOI:** 10.1186/cc14677

**Published:** 2015-09-28

**Authors:** Ana Maria Cavalheiro, Debora S da S Alves, Dejanira Aparecida Regagnin, Fabio Augusto C Vieira, Leonardo L Rocha, Maria Teresa Ap S Odierna, Maura Cristina dos Santos, Neide M Lucino

**Affiliations:** 1 Hospital Albert Einstein, Morumbi, São Paulo, São Paulo, Brazil

## Introduction

The risk factors described associated with urinary tract infection (UTI) during urethral catheterization are the colonization of the urinary tissue during evaluation. The incidence of UTI is directly related to the duration of the catheterization, and, after considering this ever-present factor in multivariate analysis, it is the main and most important risk factor for UTI. Thus, a simple noninvasive method for determining the volume of urine in the bladder would be very well received. As such, it is possible for properly trained nursing staff to use ultrasound as a vehicle for the evaluation of residual urinary volume, and Brazilian institutions have the potential to gain much based on surveys already conducted.

## Objective

To analyze the duration of time Foley catheters are used and then replace with use of ultrasound scanning to evaluate residual urine volume.

## Methods

This is a quantitative study, assessed by the ethics committee of Albert Einstein Israelita Hospital, protocol number 2267601380000071. In the first phase, 20 nurses were allowed to evaluate the residual urine volume with ultrasound and create proper care guidelines. After being taught these guidelines, nurses were allowed to remove Foley catheters from patients showing no signs of diuresis for at least 4 hours. The resulting identification of urinary volume was communicated to the attending physician and subsequent actions were determined by him. The information was recorded in a manner established in advance with volume data, volume drained, medical procedure, gender, age, reason for admission, and duration of time with indwelling bladder catheter all evaluated.

## Results

An ultrasound evaluation of residual urine volume after catheterization was performed on 94 patients in the ICU. Thirty-one of these were surgical patients and 63 were clinical patients. Thirty-six were female and 58 were male. The Foley catheter was used for an average period of 3 days (DP 1.2). The average time for the nursing team to evaluate residual urine volume was 6 hours (DP 2.3). Fifty-eight out of the 94 patients studied exhibited spontaneous diuresis after mechanical stimulation such as a change in position on the bed, use of cold or warm bags, and stimuli from the ultrasound transducer during examination and abdominal massage. Foley catheters were used in 12 patients who had more than 1000 ml urine retention 4 hours after removal of the catheter. It was recommended to use the Nelaton catheter in 28 patients with an average urinary volume of 300 ml. Of the 94 patients studied, none exhibited signs of UTI during their hospital stay.

## Conclusion

The results of this study show that the use of ultrasound as a tool for nurses to reduce Foley catheters in critically ill patients is an effective strategy to avoid ICU. Ultrasound is an effective nursing tool that safely and efficiently assesses urine volume within the bladder without the need for catheter insertion.

